# Feasibility of Short-Period, High-Dose Intravenous Methylprednisolone for Preventing Stricture after Endoscopic Submucosal Dissection for Esophageal Cancer: A Preliminary Study

**DOI:** 10.1155/2017/9312517

**Published:** 2017-07-30

**Authors:** Jun Nakamura, Takuto Hikichi, Ko Watanabe, Masaki Sato, Katsutoshi Obara, Hiromasa Ohira

**Affiliations:** ^1^Department of Gastroenterology, Fukushima Medical University School of Medicine, 1 Hikarigaoka, Fukushima City, Fukushima, Japan; ^2^Department of Endoscopy, Fukushima Medical University Hospital, 1 Hikarigaoka, Fukushima City, Fukushima, Japan; ^3^Department of Advanced Gastrointestinal Endoscopy, Fukushima Medical University, 1 Hikarigaoka, Fukushima City, Fukushima, Japan

## Abstract

**Objective:**

A wide mucosal defect after endoscopic submucosal dissection (ESD) for esophageal cancer is associated with increased risk of stricture. This study was conducted to evaluate the feasibility of short-period, high-dose intravenous methylprednisolone administration (steroid pulse therapy) in preventing post-ESD esophageal stricture.

**Methods:**

This prospective study examined 13 lesions in 11 consecutive patients with esophageal squamous cell carcinoma who underwent ESD that involved three-quarters or more of the circumference of the esophagus or who had a longitudinal resected specimen diameter of ≥5 cm. Steroid pulse therapy was initiated the day after ESD and continued for 3 consecutive days. The primary endpoint was the stricture rate after ESD. Secondary endpoints were adverse events (AEs) associated with steroid pulse therapy, time until the development of stricture, and the frequency and duration of endoscopic balloon dilation (EBD).

**Results:**

The stricture rate was 54.5% (6/11). The median time until stricture development was 15 days. The median number of EBD sessions required was 2.5. The median duration of EBD was 14.5 days. AEs related to steroid pulse therapy and postprocedure complications were not observed.

**Conclusion:**

No preventive effect of the stricture after esophageal ESD by steroid pulse therapy was found, although the therapy was administered safely.

## 1. Introduction

Endoscopic submucosal dissection (ESD), developed for treating early esophageal cancer, is associated with favorable long-term outcomes [1]. The primary purpose of ESD is to perform reliable *en bloc* resection of lesions, which contributes to reduced local recurrence [2]. However, because of the anatomically narrow luminal structure of the esophagus, ESD is associated with a risk of esophageal stricture resulting from the postoperative ulcer healing process. The reported risk factors for post-ESD esophageal stricture include a mucosal defect that covers more than three-quarters of the circumference (68–90%) [1, 3, 4] and a tumor diameter of ≥5 cm (28%) [5]. Treatment of the esophageal stricture requires frequent and long-term courses of endoscopic balloon dilation (EBD) [6], which impair the patient's quality of life (QOL). Therefore, postoperative stricture is an esophageal ESD complication that should be avoided [7].

Steroid injections to the post-ESD mucosal defect [8] and oral steroid therapy [9] have been used to prevent stricture after esophageal ESD. However, inadvertent injection into the muscle layer can result in delayed perforation [10]. Additionally, oral steroid therapy requires long-term administration of steroids over several months. It is therefore associated with increased risks of infection and the onset or worsening of diabetes [9]. In patients with collagen diseases, for so-called steroid pulse therapy, high-dose steroids are infused intravenously over a short period [11]. Because the treatment period lasts for only a few days and because it requires no endoscopic technique, it presents a reduced risk of complications such as infection and diabetes. This therapy is therefore regarded as a simple and safe means by which to administer steroids. This study was aimed at the prospective evaluation of the feasibility of steroid pulse therapy for preventing post-ESD esophageal stricture in patients who are at a high risk of postoperative stricture.

## 2. Patients and Methods

### 2.1. Patients

Subjects were chosen from patients with squamous cell carcinoma of the esophagus who underwent ESD at Fukushima Medical University Hospital between October 2011 and May 2014. Patients who met the following eligibility criteria were selected and prospectively included in this study after the completion of ESD: (1) patients with a post-ESD mucosal defect that involved three-quarters or more of the esophageal circumference or with a longitudinal resected specimen size of ≥5 cm, with or without multiple esophageal lesions; (2) those with well or moderately differentiated squamous cell carcinoma, with a preoperative invasion depth not exceeding the mucosal layer; (3) no evidence of metastasis to the lymph nodes or other organs on chest and abdominal CT scans; (4) no prior history of chemotherapy, radiotherapy, or surgery for esophageal cancer; (5) an Eastern Cooperative Oncology Group performance status of 0 or 1; (6) age ≥ 20 years; (7) preserved liver and renal function (AST ≤ 100 IU/L, ALT ≤ 100 IU/L, serum creatinine ≤ 2.0 mg/dL); and (8) no current or prior history of hepatitis B infection (negative for HBs antigen, HBc antibody, and HBe antigen). Patients were excluded if they met any of the following criteria: (1) perforation or infection after ESD; (2) an active gastric or duodenal ulcer; (3) a history of tuberculosis; (4) poorly controlled diabetes; (5) receiving continuous systemic steroid therapy; (6) receiving continuous systemic treatment with nonsteroidal anti-inflammatory drugs; (7) unable to stop taking an antithrombotic agent; (8) a concomitant psychiatric disease or symptoms that precluded participation in the study; or (9) were considered by the study investigator to be inappropriate for inclusion in this study for any other reason.

This study was conducted with approval from the ethics committee of Fukushima Medical University (Approval no. 1557), and written informed consent was obtained from all the patients. This study was registered with the University Hospital Medical Network Clinical Trial Registry (UMIN-CTR) (UMIN000010878).

### 2.2. ESD Procedure

ESD was performed in an operating room with the patient positioned in the left lateral position and managed by an anesthesiologist with endotracheal intubation. A single-channel endoscope with forward water supply function (GIF-Q260J; Olympus Medical Systems Corp., Tokyo, Japan) was used with carbon dioxide insufflation. After the extent of the lesion was determined by iodine staining, a dual knife (KD-650 L; Olympus Medical Systems Corp., Tokyo, Japan) was used to mark around the lesion with a 5 mm margin. The VIO300D high-frequency generator (ERBE Elektromedizin, Tübingen, Germany) was used. A 0.4% hyaluronic acid solution (MucoUp; Johnson and Johnson K.K., Tokyo, Japan) was then injected into the submucosa with a 25 G needle (ImpactFlow; TOP Corp., Tokyo, Japan) followed by a mucosal incision with a dual knife. Submucosal dissection was performed with a dual knife or an SB knife Jr (MD-47703; Sumitomo Bakelite Co. Ltd., Tokyo, Japan). Bleeding and blood vessels were appropriately treated by coagulation using hemostatic forceps (Coagrasper; FD-410LR; Olympus Medical Systems Corp., Tokyo, Japan). After the ESD procedure was completed and the absence of perforation on the mucosal defect was confirmed, the endoscope was removed.

### 2.3. Study Protocol

**Figure 1:**
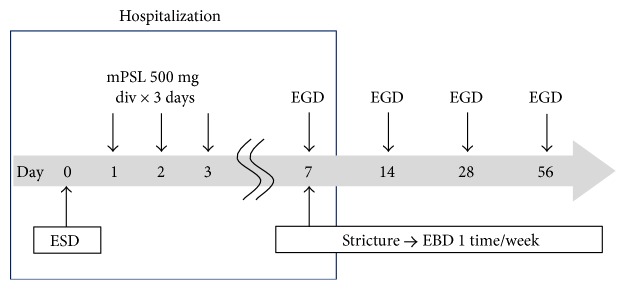
Study protocol. An intravenous infusion of methylprednisolone (500 mg/day) was initiated on the day after ESD and was continued for 3 consecutive days. On postoperative day (POD) 7, an endoscopy was performed, and the patient was discharged from the hospital. Endoscopy was also performed on PODs 14, 28, and 56 and in the event of dysphagia. Patients who were found to have stricture by endoscopy were required to undergo weekly EBD until the stricture was resolved. ESD: endoscopic submucosal dissection; EGD: esophagogastroduodenoscopy; EBD: endoscopic balloon dilatation; mPSL: methylprednisolone; div: drip intravenous infusion.

On the day after ESD, patients underwent blood testing and a chest X-ray. After no evidence of pneumonia or severe inflammatory responses was confirmed, written informed consent was provided, and the patients were enrolled in the study. Starting on the day after ESD, the patients were administered an intravenous infusion of 500 mg of methylprednisolone sodium succinate dissolved in 100 mL of saline over 1 hour for 3 consecutive days (Figure 1). This is the method used in our hospital for patients with collagen diseases, such as systemic lupus erythematosus, and is known as steroid pulse therapy. After starting steroid pulse therapy, it was discontinued if perforation or infection occurred. Although no local/oral steroid or antifungal trimethoprim-sulfamethoxazole was used prophylactically, the administration of a proton pump inhibitor was initiated on the day after ESD and was continued for 28 days.

Endoscopy was performed 7, 14, 28, and 56 days after ESD and in the event of dysphagia. Esophageal stricture was defined as the failure of the passage of a 9.9 mm endoscope (GIF-Q260J). Stricture was treated by EBD. EBD was performed with a CRE balloon (Boston Scientific Corp., Tokyo, Japan) once weekly on an outpatient basis. Balloons with diameters of 10 to 15 mm were selected, depending on the severity of the stricture, and each dilation procedure was continued until the stricture was sufficiently dilated to allow the passage of a 9.9 mm scope.

The primary endpoint was the rate of stricture after esophageal ESD. Secondary endpoints were the rate of adverse events (AEs) associated with steroid pulse therapy, the time until stricture development in the stricture cases, the frequency and duration of EBD, and the complications of EBD. The AEs associated with steroid pulse therapy were defined as infection and perforation after steroid administration. Infection was defined as a fever higher than 38°C with an infectious focus. The location, invasion depth, and macroscopic type of the lesions were described in accordance with the Japanese Convention for the Handling of Esophageal Cancer, Tenth Edition [12].

### 2.4. Statistical Analysis

The variables measured in this study are expressed as the mean, standard deviation, median, and/or range. Treatment outcomes were analyzed using the chi-square test and Student's *t*-test. Differences were considered significant for a *p* value of <0.05. All of the statistical analyses were performed with software (Statcel 3; OMS Inc., Tokorozawa, Japan).

## 3. Results

### 3.1. Patient Characteristics

**Figure 2:**
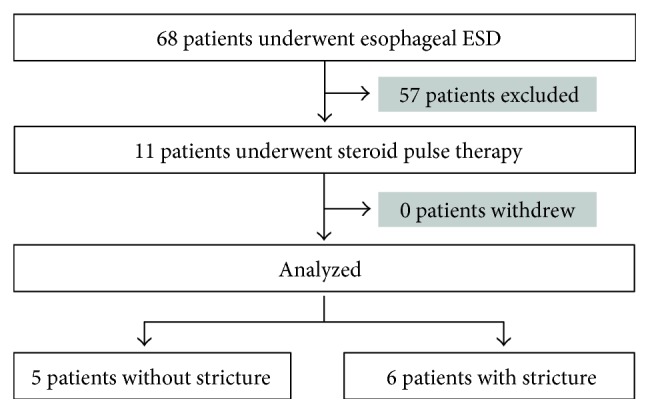
Flow chart of this study.

Of the 68 patients who underwent ESD for esophageal cancer during the period defined above, 11 patients with 13 lesions who met the eligibility criteria and did not meet any of the exclusion criteria were included in this study (Figure 2). The patient demographics are presented in Table 1. In one patient, 3 lesions were collectively resected as a single entity. The proportion of the post-ESD mucosal defect relative to the esophageal circumference was classified as follows: one-half to less than three-quarters of the circumference in 2 patients; three-quarters to less than seven-eighths of the circumference in 3 patients; and seven-eighths or more of the circumference in 6 patients. No patients had entire circumferential defects. The two patients who had defects comprising one-half to less than three-quarters of the circumference were included in the study because they had a longitudinal resected specimen size of ≥5 cm.

### 3.2. Treatment Outcomes and Adverse Events

**Figure 3:**
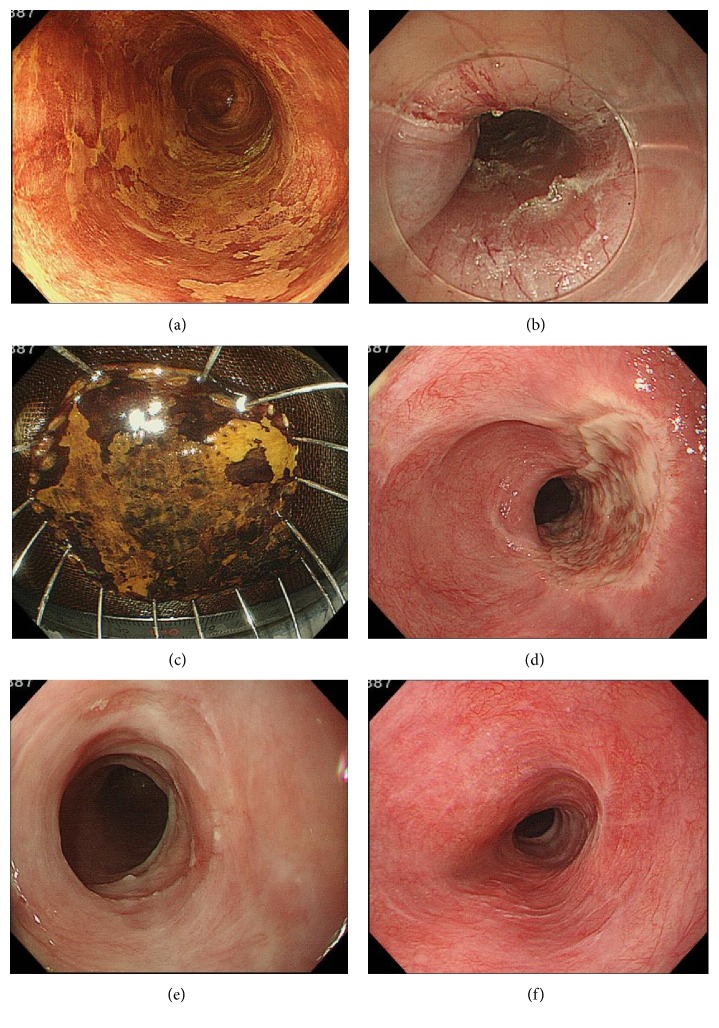
Endoscopic images of a representative patient without stricture after steroid pulse therapy. (a) An image of the esophagus stained with iodine immediately before ESD, revealing a type 0–IIc squamous cell carcinoma in the middle thoracic esophagus, is shown. (b) An image obtained immediately after ESD, demonstrating no perforation or exposed muscle layer after *en bloc* resection, is shown. The mucosal defect involved more than three-quarters but less than seven-eighths of the esophageal circumference. (c) An ESD-resected specimen stained with iodine is shown. The longitudinal resected specimen size was 53 mm. (d) An image captured 14 days after ESD is shown. The ulcer base remained covered by white moss. The lumen was slightly narrowed, but it allowed the passage of an endoscope. (e) An image obtained 56 days after ESD is shown. The ulcer was almost completely epithelialized, but it allowed the passage of an endoscope. (f) An image obtained 7 months after ESD showing complete epithelialization of the post-ESD ulcer without stricture is shown.

The post-ESD stricture rate was 54.5% (6/11). For the cases of mucosal defect that covered more than three-quarters of the circumference, the stricture rate was 66.7% (6/9). Moreover, the rate of stricture in cases of mucosal defect that covered more than seven-eighths of the circumference was 83.3% (5/6). The breakdown of the patients without stricture is presented in Table 2. The defects in these 5 patients were the following: 2 patients had defects that were between one-half and three-quarters of the circumference, 2 patients had defects that were between three-quarters and seven-eighths of the circumference, and 1 patient had a defect that was seven-eighths or more of the circumference, with a median longitudinal resected specimen size of 52 mm among the 5 patients (range, 50–111 mm). Endoscopic images of a patient without stricture (case 4) are presented in Figure 3.

**Figure 4:**
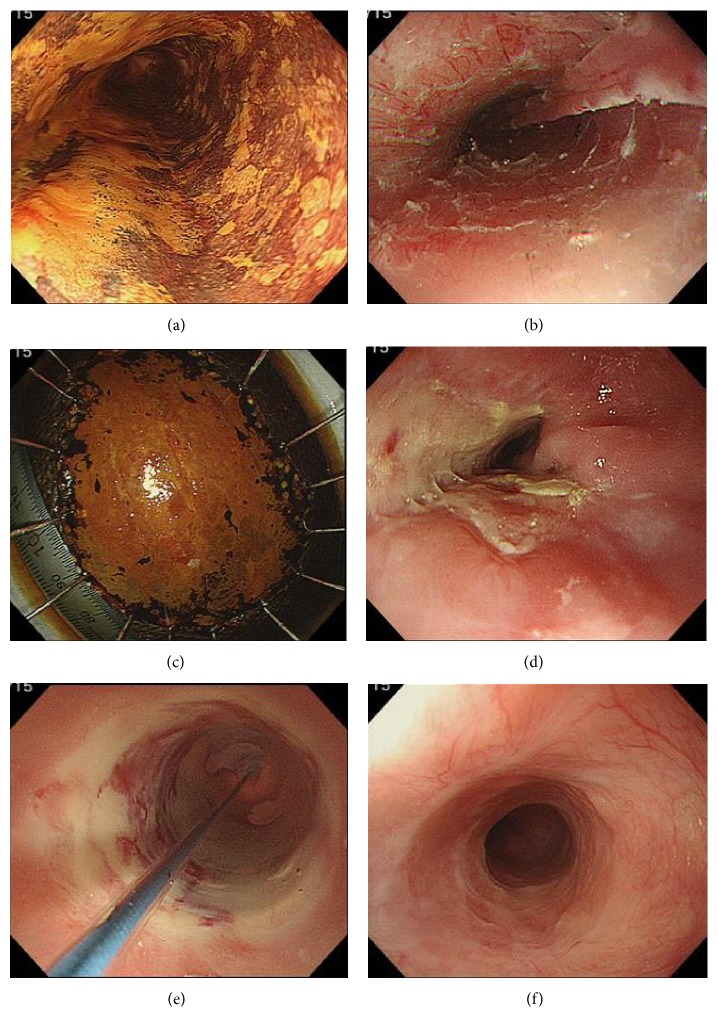
Endoscopic images of a representative patient with stricture after steroid pulse therapy. (a) An image of the esophagus stained with iodine immediately before ESD showing a type 0–IIc squamous cell carcinoma in the middle thoracic esophagus is shown. (b) An image obtained immediately after ESD showing no perforation or exposed muscle layer after *en bloc* resection is shown. The mucosal defect involved greater than seven-eighths of the esophageal circumference. (c) An ESD-resected specimen stained with iodine is shown. The longitudinal resected specimen size was 70 mm. (d) An image obtained 14 days after ESD is shown. The patient experienced dysphagia and was endoscopically found to have stricture that required endoscopic balloon dilation (EBD). (e) An image obtained 28 days after ESD is shown. An endoscope could not be passed through the esophagus, and the patient underwent additional EBD sessions. In all, 6 EBD sessions were ultimately performed. (f) An image obtained 6 months after ESD showing complete epithelialization of the post-ESD ulcer without stricture is shown.

The breakdown of the 6 patients with post-ESD stricture is presented in Table 3. Of these, 1 patient had a defect that was between three-quarters and seven-eighths of the circumference, and 5 patients had defects that were seven-eighths or more of the circumference, with a median longitudinal resected specimen size of 50.0 mm among the 6 patients (range, 40–70 mm). All of the stricture patients underwent EBD (Table 4), with a median time until stricture development of 18 days (range, 14–21 days), a median of 2.5 required EBD sessions (range, 1–6 sessions), and a median EBD duration of 14.5 days (range, 1–36 days). Endoscopic images of a stricture patient (case 3) are presented in Figure 4.

A comparison between the nonstricture and stricture cases is presented in Table 5. The stricture cases tended to have wider post-ESD defects, but no significant differences were found.

No AE associated with steroid pulse therapy was observed. Moreover, EBD-related complications such as perforation and bleeding were not observed.

## 4. Discussion

This study is the first to demonstrate the feasibility of steroid pulse therapy in the prevention of postoperative esophageal stricture after ESD in esophageal cancer patients. In post-ESD mucosal defects that involve more than three-quarters of the circumference, Ono et al. [1] reported that stricture occurred in 90% of patients following ESD, and Katada et al. [4] reported that the stricture rate was 68% after endoscopic mucosal resection. Moreover, Yamashina et al. [5] reported that the rate of stricture after ESD with a tumor size of 5 cm or greater (28%) was significantly increased compared with that of tumors that were less than 5 cm (10%). Therefore, when we started this study, a longitudinal resected specimen size of ≥5 cm was considered a risk of stricture to a similar extent as a defect that was more than three-quarters of the circumference of the esophageal lumen. However, the most important characteristic of the stricture after esophageal ESD is the circumference of post-ESD mucosal defects. In this study, the prevention effect of steroid pulse therapy for patients with mucosal defects that involved more than three-quarters of the circumference was 33.3%. Moreover, the prevention effect for the patients with a mucosal defect that covered more than seven-eighths of the circumference was only 16.3%. Therefore, we were unable to demonstrate that steroid pulse therapy prevented post-ESD stricture. However, we could show the safety of steroid pulse therapy after esophageal ESD.

During wound healing, steroids inhibit the synthesis of collagen fibers, among other tissue components. Steroids also promote the degradation of collagen fibers and exert an anti-inflammatory effect. Miyashita et al. [13] performed a histopathological evaluation of benign esophageal stricture and found that acute esophagitis characterized by fibrosis and marked inflammatory cell infiltration was a cause of the stricture. It is therefore expected that the administration of steroids immediately after ESD, which triggers inflammation, will prevent esophageal stricture by controlling the development of acute inflammation. Nonaka et al. [14] administered injections of triamcinolone acetonide (TA) to a mucosal defect after an entire circumferential esophageal ESD in pigs. Then, they reported that the steroid prevented stricture by inhibiting the proliferation of myofibroblasts and inducing the irregular arrangement and morphology of myofibroblasts.

The reported methods of steroid administration intended to prevent post-ESD esophageal stricture have included injections to the mucosal defect, oral administration, and combined use with EBD [8, 9, 15–19]. Hashimoto et al. [8] performed injections of TA to post-ESD mucosal defects that involved at least three-quarters of the esophageal circumference. They reported a significantly reduced stricture rate in patients treated with TA injection (19%) compared with those not treated with TA (75%). The mean number of EBD sessions required was also significantly reduced in TA-treated patients (1.7 sessions) compared with those not treated with TA (6.6 sessions). Hanaoka et al. [15] conducted a prospective study of TA injections in patients with post-ESD mucosal defects that involved at least three-quarters of the esophageal circumference using a historical control group of patients not treated with TA. They performed single injections of TA immediately after ESD and reported a significantly reduced stricture rate in patients treated with TA (10%) compared with the nontreated control group (66%). The median number of required EBD sessions was also significantly reduced in the TA-treated group (0 sessions) compared with the control group (2 sessions). In contrast, Yamaguchi et al. [9] proposed the systemic administration of oral steroids. They administered oral prednisolone to a total of 19 patients with post-ESD mucosal defects that involved at least three-quarters of the esophageal circumference, including 3 patients with entire circumferential defects, at a starting dose of 30 mg/day beginning 3 days after surgery followed by dose reduction in increments of 5 mg every 7 days for a total of 8 weeks. They reported a stricture rate of 5.3%, and the mean number of EBD sessions required in stricture cases was 1.7. The long-term use of steroids carries risks of increased susceptibility to infection because of compromised immunity, diabetes, or peptic ulcer disease. Moreover, Kataoka et al. [20] recently reported that short-term (3 weeks) and low-dose oral steroid administration prevented post-ESD esophageal stricture in 17.6% of the examined cases. Although these findings suggest the efficacy of steroids in preventing post-ESD esophageal stricture, controversy persists related to their proper usage and dosage as well as the timing and duration of administration. There has also been demand for alternative therapies that are simpler and have lower risks of AEs.

We then focused on steroid pulse therapy, which has been used to treat patients with collagen diseases. Although no report of the relevant literature describes a study addressing the use of steroid pulse therapy after endoscopic treatment, Morikawa et al. [21] demonstrated the efficacy of high-dose intravenous steroid therapy with methylprednisolone in preventing benign esophageal stricture in pediatric patients who were resistant to steroid injection. They also reported the use of high-dose intravenous steroid therapy with dexamethasone administered immediately after balloon dilation and found that steroids prevented stricture by inhibiting the early development of inflammation. Steroid pulse therapy offers three advantages. First, intravenous administration provides a rapid increase in blood concentration. Therefore, it can be expected to ensure the action of the steroids in the early stages of inflammation. Second, a shorter period of steroid treatment is required compared with oral administration. Pulse therapy only requires a 3-day treatment period and thus has a low risk of complications. Therefore, we did not use antifungal prophylaxis in the present study. Third, unlike injections to a post-ESD mucosal defect, injections to a fragile ulcer surface or thin muscle layer can be avoided. Inadvertent injections of a steroid into the muscle layer can result in delayed perforation [10].

Post-ESD esophageal stricture has been reported to occur between 2 and 4 weeks after surgery [3, 7, 14]. In the present study, the median postoperative time until the development of stricture in 6 cases was 15 days, which is consistent with the previous reports. This finding suggested that although steroid pulse therapy is effective in preventing stricture in the acute postoperative phase by suppressing inflammation, additional strategies are necessary to prevent stricture, which can occur later. Sato et al. [16] reported stricture prevention by the addition of an early treatment with oral steroids after balloon dilatation, suggesting the potential efficacy of sequential steroid pulse therapy followed by maintenance steroid therapy for the continued suppression of stricture formation during the ulcer healing process.

The limitations of this study were the following. First, this study, which was conducted as a pilot study at a single institution, included only a few patients. The primary endpoint was limited by the small number of patients included in the study. Second, this study was not a randomized controlled trial (RCT). Finally, steroid pulse therapy alone was insufficient for managing mucosal defects that involved seven-eighths or more of the esophageal circumference. Therefore, steroid pulse therapy combined with local or oral steroid administration should be evaluated in a multicenter RCT.

## 5. Conclusion

This report is the first to evaluate the feasibility of steroid pulse therapy in preventing post-ESD esophageal stricture. However, we were unable to demonstrate a preventive effect of the stricture after esophageal ESD by steroid pulse therapy. Therefore, further studies must be conducted to elucidate the mechanism by which steroid pulse therapy prevents stricture and the potential for combination therapies.

## Figures and Tables

**Table 1:** Patient characteristics.

Age, median (range), y	71 (57–83)
Male/female	11/0
Tumor location (Ut/Mt/Lt)	0/9^∗^/4
Macroscopic type (IIc/IIb)	11^∗^/2
Depth of tumor invasion (EP·LPM/MM·SM1/SM2)	10^∗^/2/1^∗^
Mucosal defect circumference (1/2–<3/4 : ≥3/4–<7/8 : ≥7/8)	2 : 3 : 6
Longitudinal resected specimen size, median (range), mm	52 (30–111^∗^)
ESD procedure time, median (range), min	89 (50–130)

^∗^Three lesions were completely resected in one piece in one patient. The lesions in this patient were located in the Mt, and the tumor depths were 2 EP and 1 SM2. Ut: upper thoracic esophagus; Mt: middle thoracic esophagus; Lt: lower thoracic esophagus; EP: epithelium; LPM: lamina propria mucosa; MM: muscularis mucosa; SM 1: slight submucosal invasion (less than 200 *μ*m from MM); SM 2: deep submucosal invasion (greater than 200 *μ*m from MM); ESD: endoscopic submucosal dissection.

**Table 2:** Nonstricture cases administered steroid pulse therapy after esophageal ESD.

Case	Sex	Age (yr)	Location	Mucosal defect circumference	Longitudinal resected specimen size (mm)	Tumor depth
1	M	75	Lt	1/2–<3/4	50	MM
2	M	60	Lt	≥3/4–<7/8	50	LPM
3^∗^	M	63	Mt	≥7/8	111	1 SM2, 2 EP
4	M	70	Mt	≥3/4–<7/8	53	EP
5	M	83	Lt	1/2–<3/4	52	EP

^∗^In case 3, three lesions were completely resected in one piece. ESD: endoscopic submucosal dissection; Mt: middle thoracic esophagus; Lt: lower thoracic esophagus; EP: epithelium; LPM: lamina propria mucosa; MM: muscularis mucosa; SM2: deep submucosal invasion (greater than 200 *μ*m from MM).

**Table 3:** Stricture cases administered steroid pulse therapy after esophageal ESD.

Case	Sex	Age (yr)	Location	Mucosal defect circumference	Longitudinal resected specimen size (mm)	Depth	Period from ESD to stricture (days)	No. of required EBDs	EBD period(days)
1	M	67	Mt	≥7/8	45	MM	21	3	14
2	M	78	Lt	≥3/4–<7/8	40	LPM	21	1	1
3	M	76	Mt	≥7/8	70	LPM	15	6	36
4	M	71	Mt	≥7/8	50	EP	15	1	1
5	M	72	Lt	≥7/8	50	EP	15	5	35
6	M	57	Mt	≥7/8	70	EP	14	2	15

ESD: endoscopic submucosal dissection; EBD: endoscopic balloon dilation; Mt: middle thoracic esophagus; Lt: lower thoracic esophagus; EP: epithelium; LPM: lamina propria mucosa; MM: muscularis mucosa.

**Table 4:** Endoscopic balloon dilation for stricture cases administered steroid pulse therapy after esophageal ESD (*n* = 6).

Period from ESD to stricture, median (range), days	15 (14–21)
No. of required EBD sessions, median (range)	2.5 (1–6)
EBD period^∗^, median (range), days	14.5 (1–36)
Complications of EBD (%)	0

^∗^From the first EBD to the final EBD. ESD: endoscopic submucosal dissection; EBD: endoscopic balloon dilation.

**Table 5:** Comparison between nonstricture and stricture cases.

	Nonstricture (7 lesions in 5 patients)	Stricture (6 lesions in 6 patients)	*p* value
Sex			
Male	5	6	
Female	0	0	
Age, mean (±SD), y	70.2 (±9.3)	70.2 (±7.5)	0.99
Tumor location			0.45
Ut	0	0	
Mt	4	4	
Lt	3	2	
Macroscopic type			0.15
IIc	5	6	
IIb	2	0	
Depth of tumor invasion			0.36
EP/LPM	5	5	
MM/SM1	1	1	
SM2	1	0	
Mucosal defect circumference			0.24
1/2–<3/4	2	0	
≥3/4–<7/8	2	1	
≥7/8	1	5	
Longitudinal resected specimen size, mean (±SD), mm	64.2 (±26.2)	54.2 (±12.8)	0.43

Ut: upper thoracic esophagus; Mt: middle thoracic esophagus; Lt: lower thoracic esophagus; EP: epithelium; LPM: lamina propria mucosa; MM: muscularis mucosa; SM 1: slight submucosal invasion (less than 200 *μ*m from MM); SM 2: deep submucosal invasion (greater than 200 *μ*m from MM).
